# Supplementary material for: Synthesis and Biological Investigation of Bile Acid-Paclitaxel Hybrids
Source: Molecules. 2022 Jan 12;27(2):471. doi: 10.3390/molecules27020471 (PMC8779069; doi:10.3390/molecules27020471)

Synthesis and Biological Investigation of Bile Acid-Paclitaxel Hybrids

Elisabetta Melloni[1#], Elena Marchesi[2#], Lorenzo Preti[3], Fabio Casciano[1,4], Erika Rimondi[1], Arianna Romani[1], Paola Secchiero[1], Maria Luisa Navacchia[5*], Daniela Perrone[2*]

1 Department of Translational Medicine and LTTA Centre, University of Ferrara, 44121 Ferrara, Italy; elisabetta.melloni@unife.it (E.M.); fabio.casciano@unife.it (F.C.); erika.rimondi@unife.it (E.R.); arianna.romani@unife.it (A.R.); paola.secchiero@unife.it (P.S.)

2 Department of Environmental and Prevention Sciences, University of Ferrara, 44121 Ferrara, Italy; mrclne@unife.it

3 Department of Chemical and Pharmaceutical Sciences, University of Ferrara, 44121 Ferrara, Italy; prtlnz@unife.it

4 Interdepartmental Research Center for the Study of Multiple Sclerosis and Inflammatory and Degenerative Diseases of the Nervous System, University of Ferrara, 44121 Ferrara, Italy

5 Institute of Organic Synthesis and Photoreactivity, Italian National Research Council, 40129 Bologna, Italy

* Correspondence: marialuisa.navacchia@isof.cnr.it (M.L.N.); prd@unife.it (D.P.)

† These authors equally contributed to the work.

Supplementary Figure S1

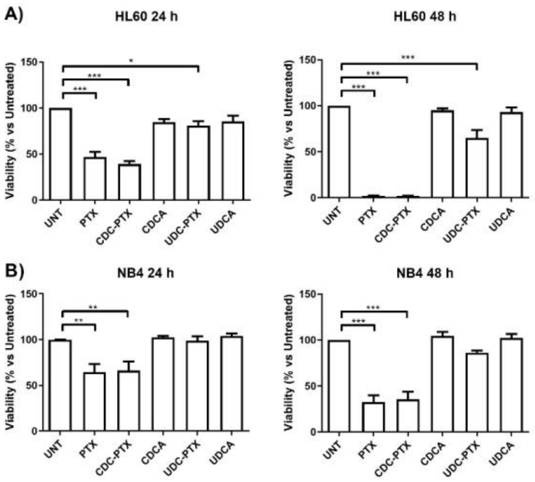

Figure S1. MTT assay for cell viability determination in HL60 (A) or NB4 (B) cells treated for 24 or 48 hours with 2 μM of each of the following compounds: PTX; CDC-PTX, CDCA, UDC-PTX or UDCA. Data are reported as mean±SEM of at least 3 independent experiments. Statistical analysis was performed by ANOVA followed by Bonferroni post-hoc test for pairwise comparisons. *, p<0.05; **, p<0.01; ***, p<0.001.

Supplementary Figure S2

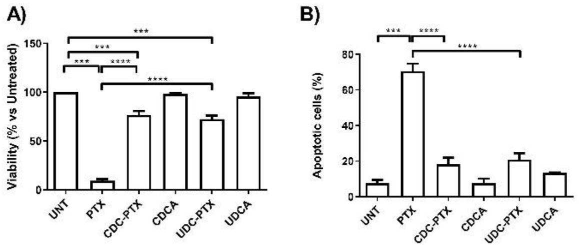

Figure S2. NIH-3T3 viability (A) and apoptosis (B) evaluation after 4 hours of treatment with 5 μM of each compound (PTX; CDC-PTX, CDCA, UDC-PTX or UDCA) followed by 72 hours of incubation with fresh media. Data are reported as mean ± SEM of at least 3 independent experiments. Statistical analysis was performed by ANOVA followed by Bonferroni post-hoc test for pairwise comparisons. ***, p<0.001; ****p<0.0001.

^{1}H-NMR of compound UDC-PTX

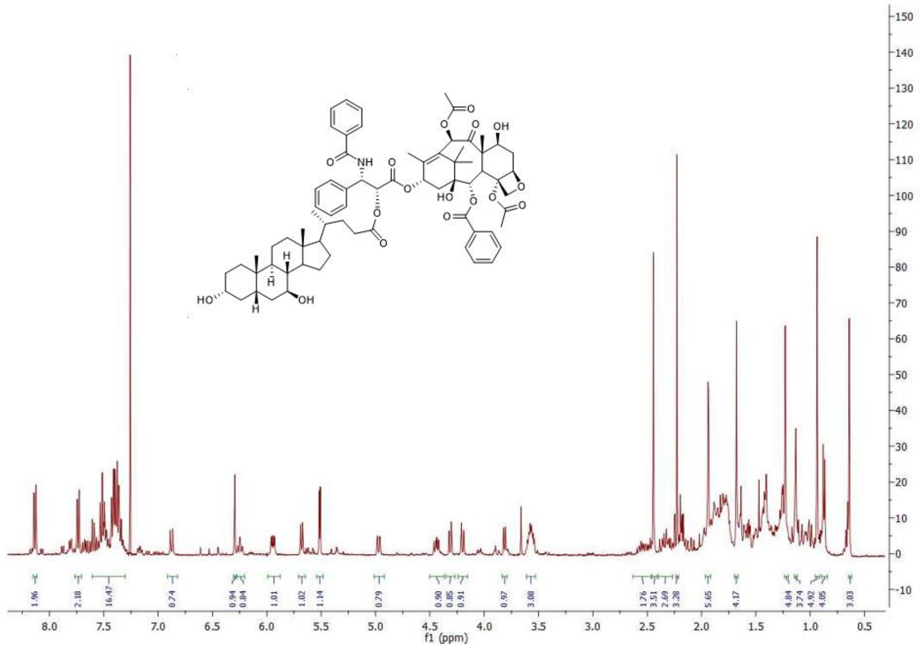

^{13}C-NMR of compound UDC-PTX

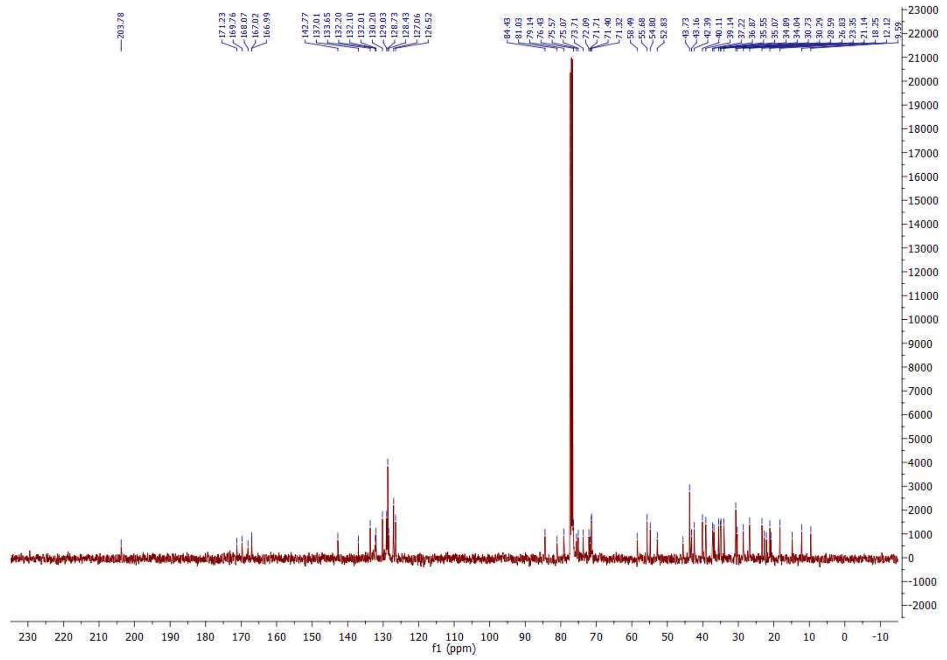

^{1}H-NMR of compound CDC-PTX

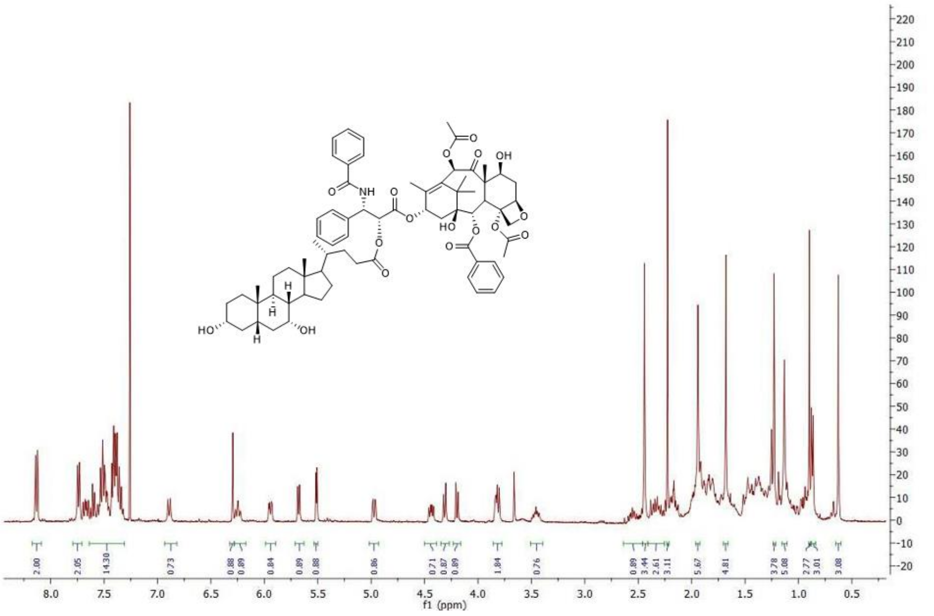

^{13}C-NMR of compound CDC-PTX

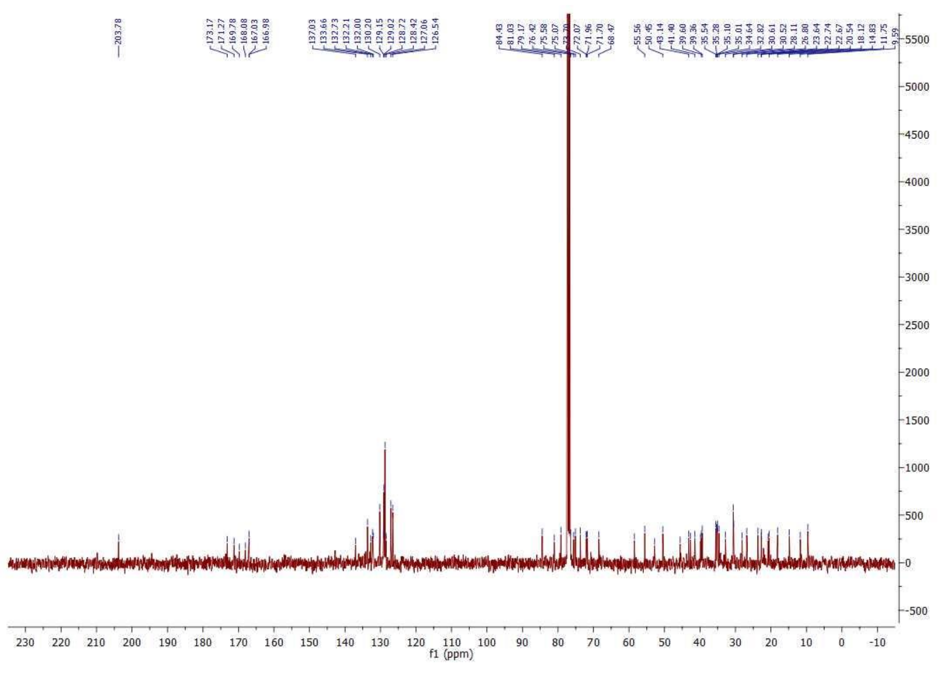

^{1}H-NMR of compound 1

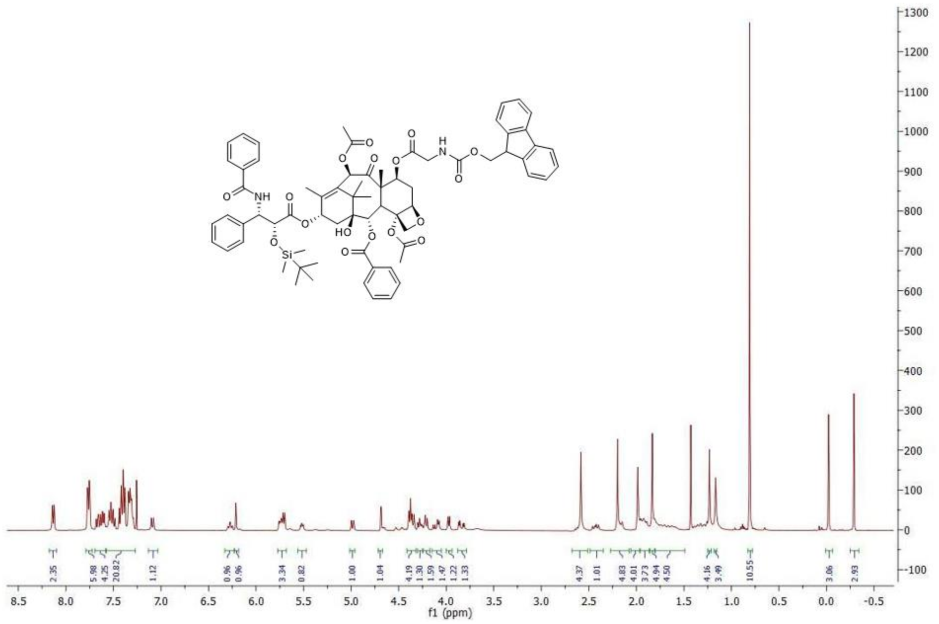

^{13}C-NMR of compound 1

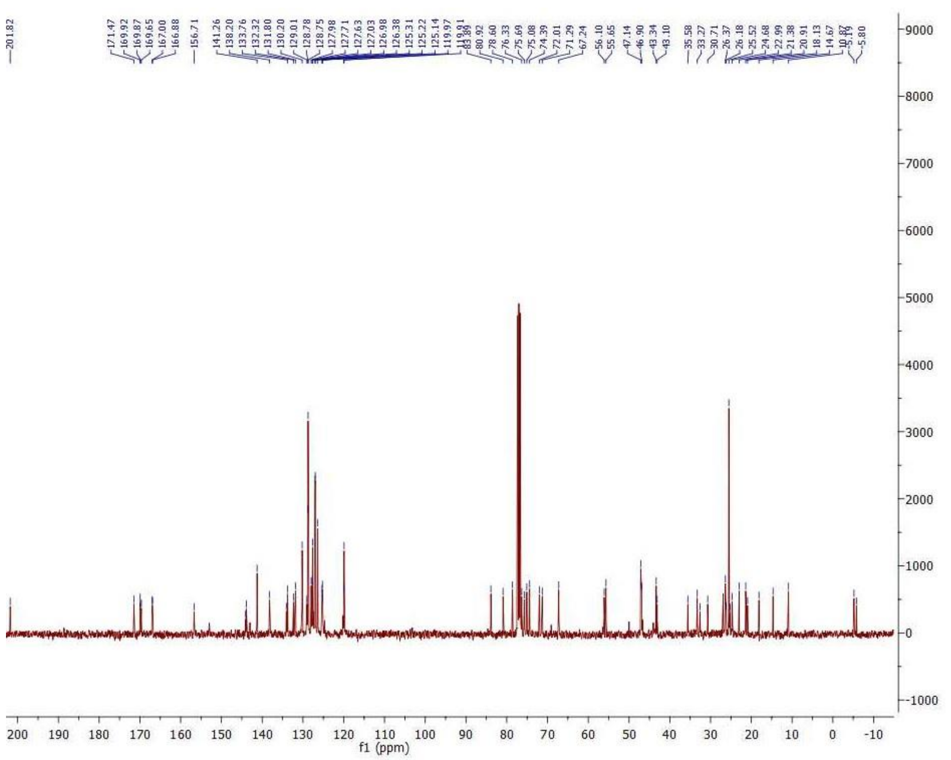

^{1}H-NMR of compound 2

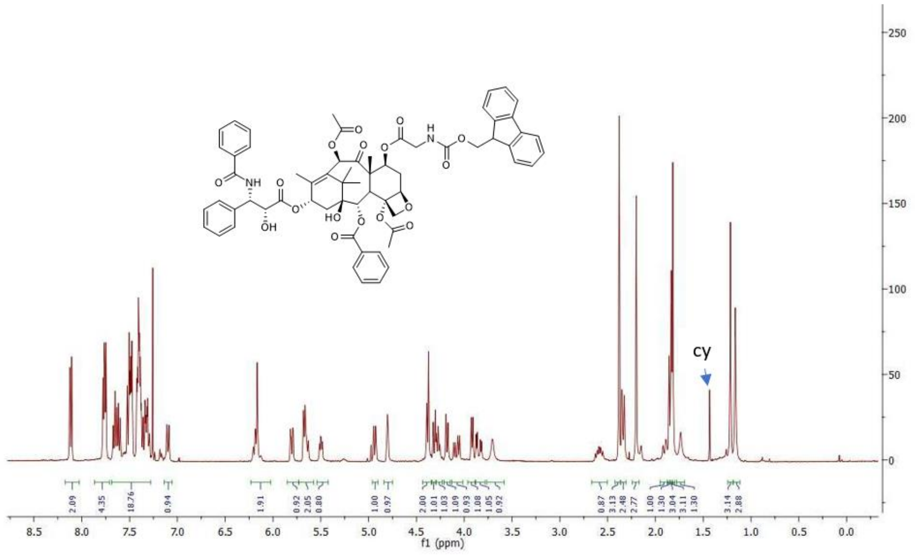

^{13}C-NMR of compound 2

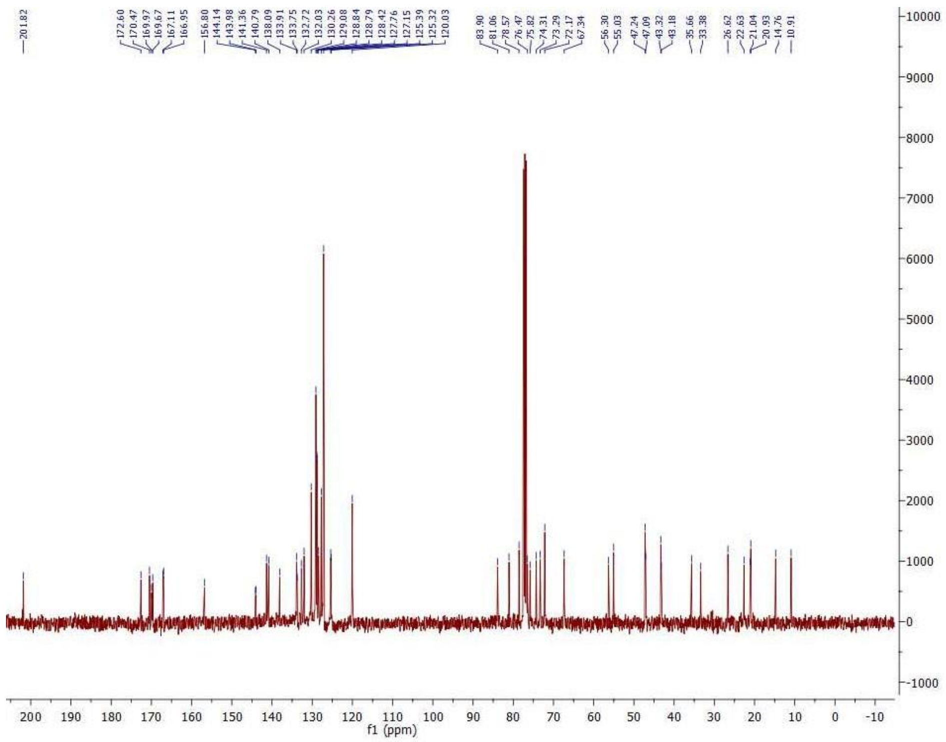

^{1}H-NMR of compound UDC-PTX-PB

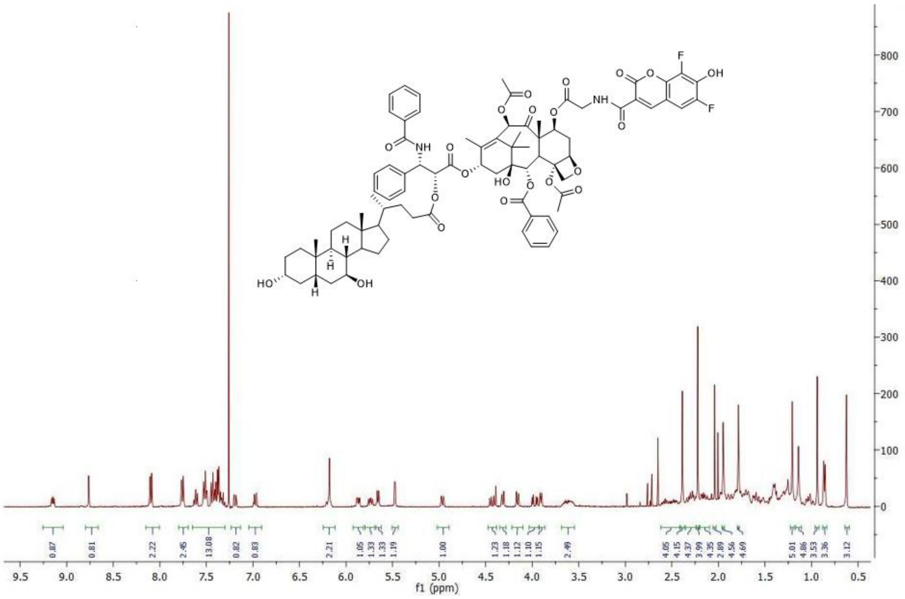

RP-HPLC chromatogram of compound UDC-PTX-PB. Rt=12.1 min

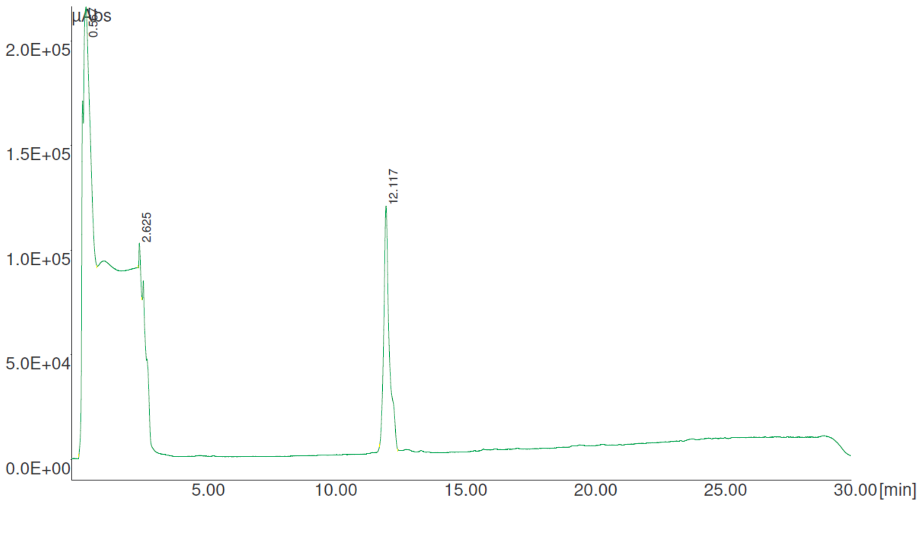

^{1}H-NMR of compound CDC-PTX-PB

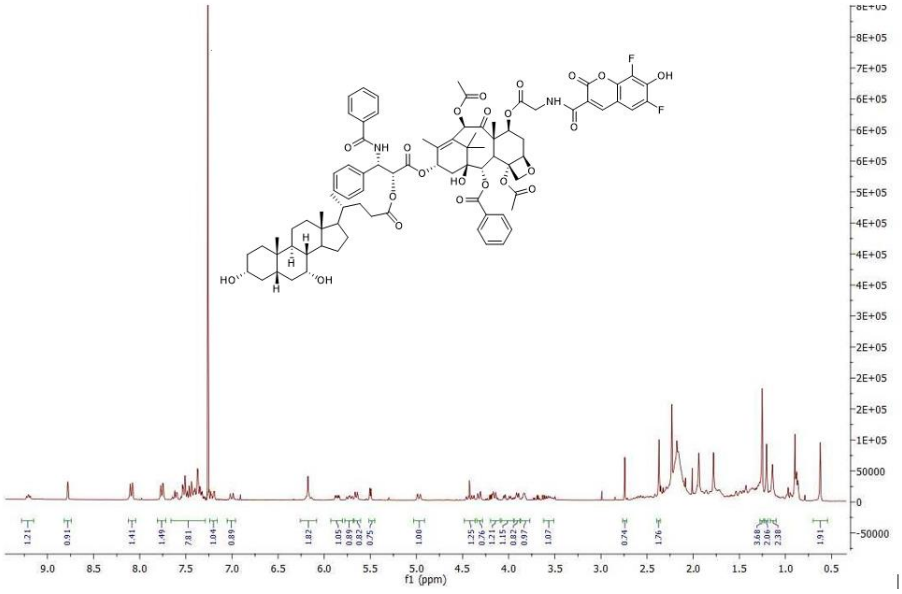

RP-HPLC chromatogram of compound CDC-PTX-PB. Rt=9.5 min

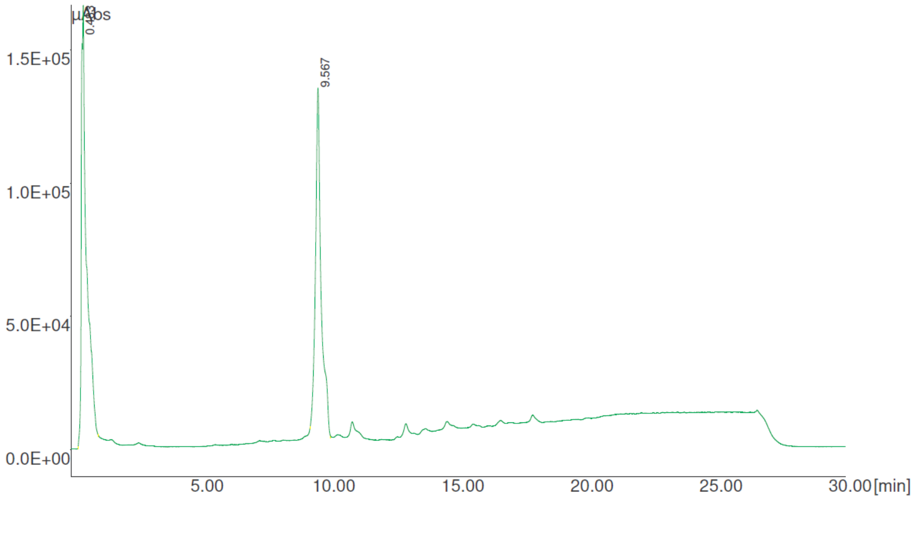

^{1}H-NMR of compound 5

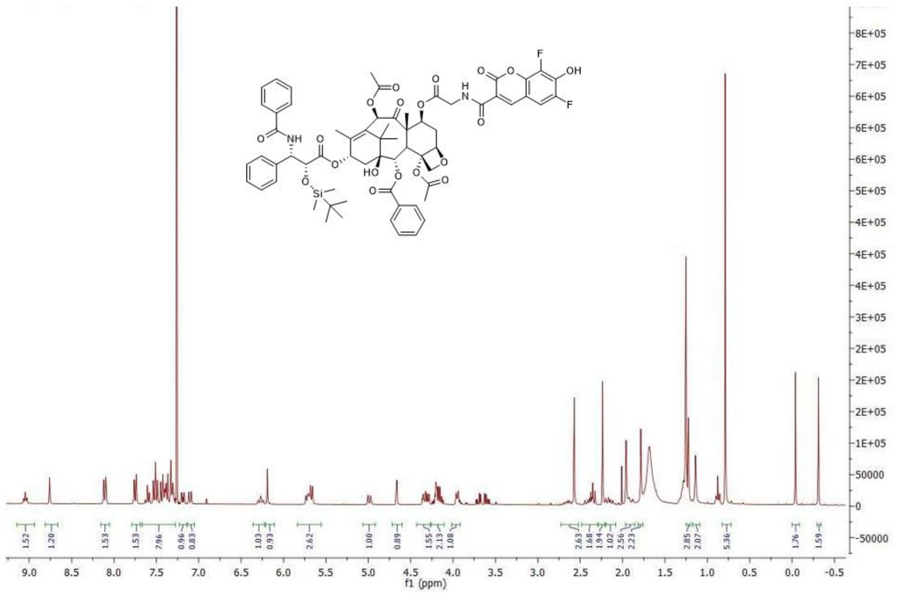

RP-HPLC chromatogram of compound 5. Rt=11.4 min

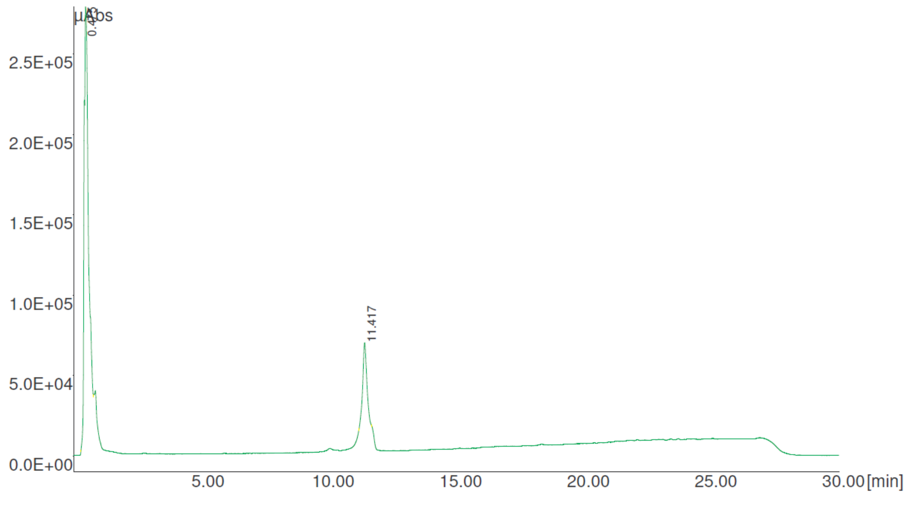

Supplement: Supplementary file 1 [file molecules-27-00471-s001.zip › molecules-1530310-supplementary.pdf]
